# Magnetic Resonance Imaging and X-Ray Imaging Properties of Ultrasmall Lanthanide Oxide (Ln = Eu, Gd, and Tb) Nanoparticles Synthesized via Thermal Decomposition

**DOI:** 10.3390/molecules30122519

**Published:** 2025-06-09

**Authors:** Shuwen Liu, Tirusew Tegafaw, Son Long Ho, Huan Yue, Dejun Zhao, Ying Liu, Endale Mulugeta, Xiaoran Chen, Hansol Lee, Dabin Ahn, Ji-ung Yang, Ji Ae Park, Ahrum Baek, Jihyun Kim, Yongmin Chang, Gang Ho Lee

**Affiliations:** 1 Department of Chemistry, College of Natural Sciences, Kyungpook National University, Taegu 41566, Republic of Korea; liushuwen0701@gmail.com (S.L.); tirukorea@gmail.com (T.T.); sonlongh@gmail.com (S.L.H.); yuehuan888@gmail.com (H.Y.); djzhao.chem@gmail.com (D.Z.); ly1124161@gmail.com (Y.L.); endexindex05@gmail.com (E.M.); tsukiyovo@gmail.com (X.C.); 2 Division of Biomedical Science, School of Medicine, Kyungpook National University, Taegu 41944, Republic of Korea; leehs9836@naver.com (H.L.); adb9504@naver.com (D.A.); 3 Division of RI-Convergence Research, Korea Institute of Radiological & Medical Science, Seoul 01817, Republic of Korea; wjy11300@kirams.re.kr (J.-u.Y.); jpark@kirams.re.kr (J.A.P.); 4 Institute of Biomedical Engineering Research, Kyungpook National University, Taegu 41944, Republic of Korea; baxun@naver.com; 5 Department of Chemistry Education, Teachers’ College, Kyungpook National University, Taegu 41566, Republic of Korea; jkim23@knu.ac.kr; 6 Department of Molecular Medicine, School of Medicine, Kyungpook National University, Taegu 41944, Republic of Korea

**Keywords:** lanthanide oxide nanoparticle, thermal decomposition, ligand exchange, imaging properties, magnetic resonance imaging, X-ray imaging

## Abstract

Owing to their 4f electrons and high atomic numbers, lanthanide (Ln) elements impart lanthanide oxide (Ln_2_O_3_) nanoparticles with excellent biomedical imaging properties. This study reports synthesis for three types of ultrasmall and monodisperse Ln_2_O_3_ nanoparticles (Ln = Eu, Gd, and Tb) via thermal decomposition in oleylamine at 280 °C, followed by ligand exchange with citric acid (CA) to produce water-dispersible, CA-grafted Ln_2_O_3_ nanoparticles with high colloidal stability. The resulting CA-grafted Ln_2_O_3_ nanoparticles had average diameters of approximately 2 nm. We characterized their physicochemical properties, including in vitro cytotoxicity, magnetic resonance imaging properties (i.e., water proton spin relaxivities), and X-ray imaging properties (i.e., X-ray attenuation).

## 1. Introduction

Advances in multimodal diagnostic imaging offer the potential for more precise and accurate disease treatment [1,2]. A key challenge is the development of imaging agents, compatible with multiple modalities such as magnetic resonance imaging (MRI), X-ray computed tomography (CT), and fluorescence imaging (FI) [3,4,5,6,7]. These techniques have complementary strengths in terms of spatial and temporal resolution, sensitivity, and contrast. For example, CT provides high-quality 3D images of hard tissues such as bone [8], whereas MRI excels in generating 3D images of soft tissues with high resolution and unlimited imaging depth [9,10]. FI is also valuable for its high sensitivity and capacity for multiplex imaging [11]. Multimodal imaging offers enhanced performance over individual techniques, including greater detection sensitivity, imaging depth, and spatial resolution, enabling more detailed and accurate disease characterization [12,13,14,15,16,17]. Therefore, increasing efforts have been devoted to developing multimodal contrast agents and imaging devices.

Nanoparticles have been widely explored as multimodal imaging agents because of their superior imaging properties compared to molecular imaging agents. Among them, lanthanide oxide (Ln_2_O_3_) nanoparticles have attracted significant attention for their multiple imaging properties, attributed to the unique 4f electronic structure and high atomic numbers of Ln elements. The 4f electrons are primarily responsible for the appreciable magnetic moments of the Ln_2_O_3_ nanoparticles at room temperature [18], and these moments remain largely unaffected by surface ligands or particle size owing to the shielding of compact 4f orbitals by 5s and 5p orbitals [19]. Therefore, ultrasmall Ln_2_O_3_ nanoparticles (<3 nm) can be synthesized without compromising magnetic properties, which are beneficial for renal clearance [20,21]. In addition, their high atomic numbers enable strong X-ray attenuation surpassing that of iodine-based molecular contrast agents.

Previous studies have reported various methods for synthesizing Ln_2_O_3_ nanoparticles. Ultrasmall Ln_2_O_3_ nanoparticles coated with D-glucuronic acid or polyacrylic acid were synthesized using a one-pot polyol method [22,23,24,25,26]. Eu- and Tb-doped nanomaterials were synthesized using solid-state techniques [27,28]. Other methods included sonochemistry [29], coprecipitation [30,31], and calcination [32,33]. However, these previous methods have several disadvantages, such as particle distributions, large sizes (>5 nm), poor water-solubility, and fluorescence quenching [34,35,36] owing to water coordination.

In this study, we employed a thermal decomposition method [37,38,39] to synthesize ultrasmall and monodispersed Ln_2_O_3_ (Ln = Eu, Gd, and Tb) nanoparticles. While this method had been used to synthesize quantum dots and 3d-transition metal oxide nanoparticles in organic solvents [37,38] as well as Ln^3+^-doped 3d-transition metal oxide nanoparticles [39], it employed oleic acid and oleylamine as solvents and capping agents [37,38]. However, only oleylamine was used in this study as its amine groups are easily exchangeable with the carboxyl groups of grafting ligands. This study aimed to synthesize ultrasmall and monodispersed citric acid (CA)-grafted Ln_2_O_3_ nanoparticles with high colloidal stability in aqueous media. Their physicochemical properties were evaluated to assess their potential as multimodal contrast agents for MRI and CT.

## 2. Results and Discussion

### 2.1. Physicochemical Properties

**Figure 1:**
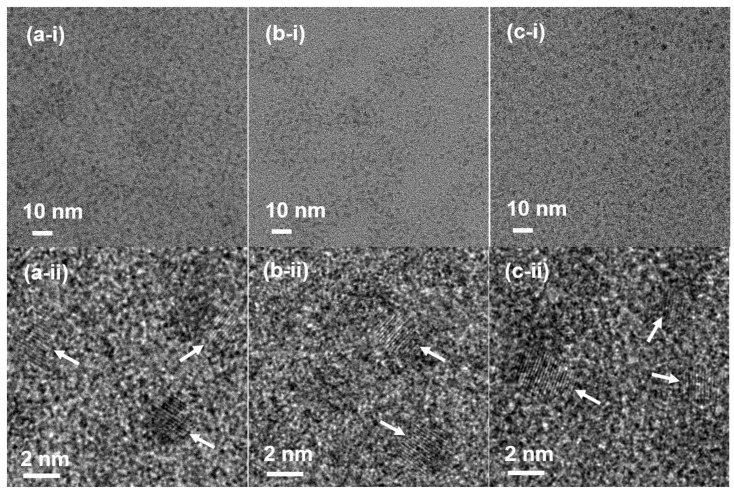
HRTEM images of CA-grafted Ln_2_O_3_ nanoparticles: Ln = (**a-i**,**a-ii**) Eu, (**b-i**,**b-ii**) Gd, and (**c-i**,**c-ii**) Tb, shown at 2 and 10 nm scales. Arrows indicate CA-grafted Ln_2_O_3_ nanoparticles.

**Figure 2:**
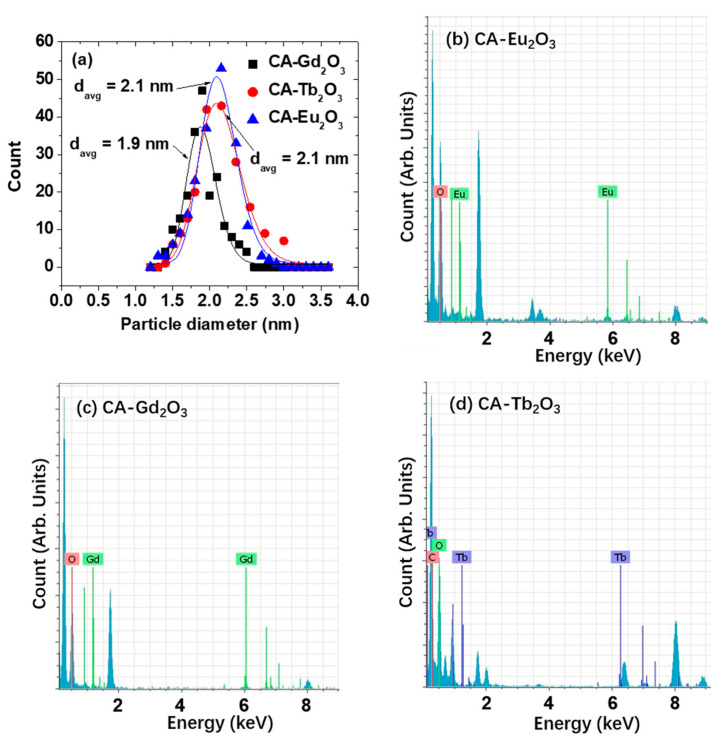
(**a**) Log-normal function fits of particle diameter distributions for CA-grafted Ln_2_O_3_ nanoparticles used to determine d_avg_. EDS spectra of CA-grafted Ln_2_O_3_ nanoparticles: Ln = (**b**) Eu, (**c**) Gd, and (**d**) Tb.

CA-grafted Ln_2_O_3_ (Ln = Eu, Gd, and Tb) nanoparticles were successfully synthesized via thermal decomposition followed by ligand exchange. High-resolution transmission electron microscopy (HRTEM) revealed particle diameters ranging from 1.1 to 3.0 nm (Figure 1a–c). The average particle diameters (d_avg_), determined by log-normal function fitting of the particle diameter distributions, were 2.1 ± 0.1 nm (Eu), 1.9 ± 0.1 nm (Gd), and 2.1 ± 0.1 nm (Tb) (Figure 2a, Table 1). Energy dispersive spectroscopy (EDS) further confirmed the presence of Eu, Gd, and Tb in the nanoparticles (Figure 2b–d).

**Figure 3:**
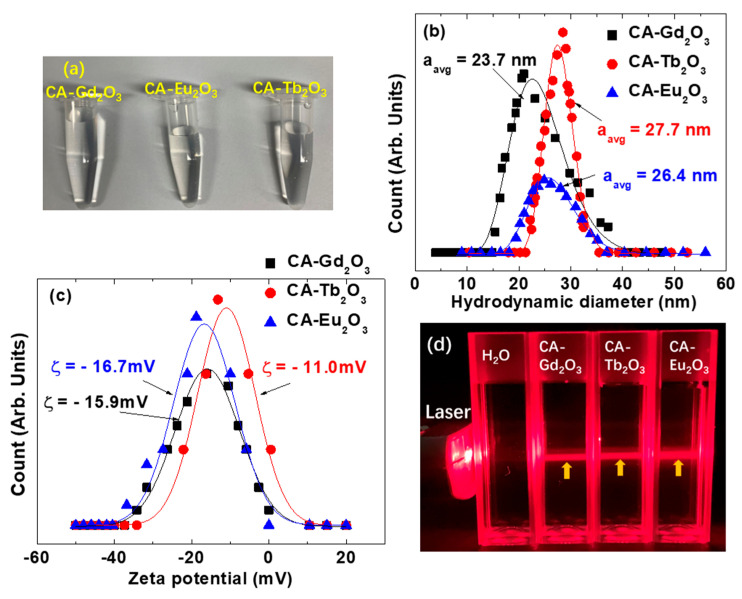
(**a**) Photographs of CA-grafted Ln_2_O_3_ nanoparticles (Ln = Eu, Gd, and Tb) dispersed in aqueous media. (**b**) Log-normal function fits to DLS data. (**c**) Zeta potential plots. (**d**) Tyndall effects confirming nanoparticle colloidal dispersion in aqueous media; arrows indicate laser scattering by the nanoparticles.

Figure 3a shows aqueous solutions of CA-grafted Ln_2_O_3_ nanoparticles (Ln = Eu, Gd, and Tb) at concentrations of ~20 mM Ln, forming transparent, well-dispersed colloidal suspensions. Their average hydrodynamic diameters (a_avg_) were 26.4 ± 1.0 nm, 23.7 ± 1.0 nm, and 27.7 ± 1.0 nm for Ln = Eu, Gd, and Tb, respectively, derived from log-normal functions fits of dynamic light scattering (DLS) data (Figure 3b, Table 1). The nanoparticles exhibited high negative zeta potentials (ζ) of −16.7 ± 0.4 mV, −15.9 ± 0.2 mV, and −11.0 ± 0.3 mV for Ln = Eu, Gd, and Tb (Figure 3c, Table 1), respectively, attributed to the carboxyl groups of CA. The Tyndall effect was observed only in the nanoparticle solutions (Figure 3d), confirming their good colloidal stability compared to triple-distilled water.

### 2.2. Crystallinity

**Figure 4:**
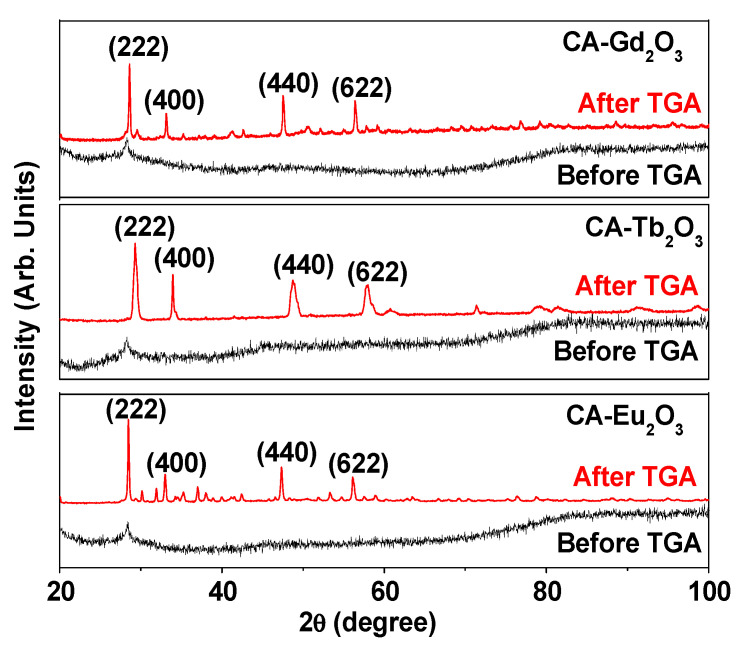
XRD patterns of CA-grafted Ln_2_O_3_ nanoparticles (Ln = Eu, Gd, and Tb) before (as-prepared) and after TGA. After TGA, all peaks corresponded to cubic Eu_2_O_3_, Gd_2_O_3_, and Tb_2_O_3_ (hkl) indices, with only the strong peaks explicitly indexed.

X-ray diffraction (XRD) patterns of the synthesized nanoparticle powders were recorded before and after thermogravimetric analysis (TGA) (Figure 4). Before TGA, the XRD patterns lacked sharp peaks because of incomplete nanoparticle crystallization from ultrasmall particle sizes [40]. However, after TGA up to 900 °C, sharp cubic Ln_2_O_3_ (Ln = Eu, Gd, and Tb) peaks appeared, indicating crystal growth. The lattice constants of TGA-treated nanoparticles were 10.853, 10.802, and 10.556 Å for Ln = Eu, Gd, and Tb, respectively, closely matching reported values of 10.863, 10.813, and 10.700 Å, respectively (Card No. Eu_2_O_3_: 03-065-3182, Gd_2_O_3_: 00-012-0797, and Tb_2_O_3_: 01-074-1986) [41].

### 2.3. Surface-Grafting Results

**Figure 5:**
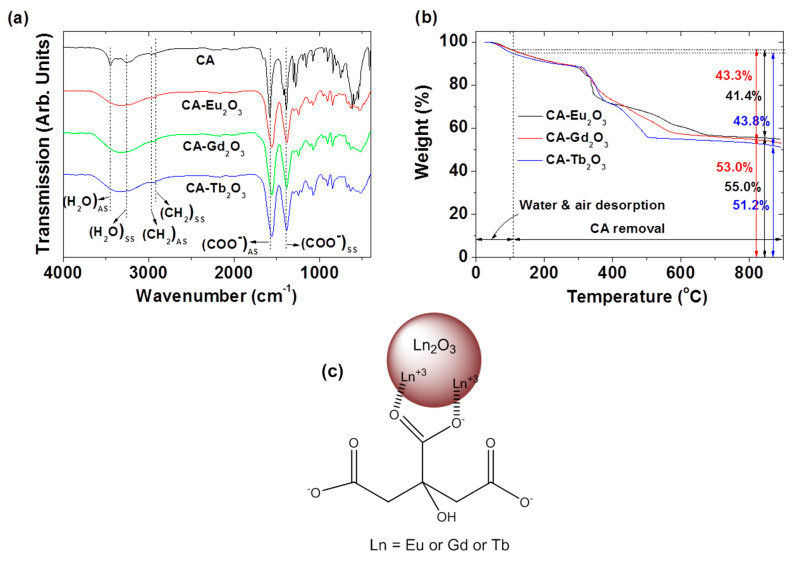
(**a**) FT-IR absorption spectra of CA and CA-grafted Ln_2_O_3_ nanoparticles (Ln = Eu, Gd, and Tb). Subscripts AS and SS indicate asymmetric and symmetric stretching, respectively. (**b**) TGA curves of CA-grafted Ln_2_O_3_ nanoparticles (Ln = Eu, Gd, and Tb). (**c**) Grafting structure of CA on Ln_2_O_3_ nanoparticle surfaces (Ln = Eu, Gd, and Tb). TGA data indicate that 40–70 CA molecules were grafter per nanoparticle (Table 1).

The surface grafting of Ln_2_O_3_ nanoparticles (Ln = Eu, Gd, and Tb) with CA was confirmed by Fourier transform-infrared (FT-IR) absorption spectra and compared with that of CA as reference (Figure 5a). The CA characteristic COO^−^ antisymmetric (1582 cm^−1^) and symmetric (1388 cm^−1^) stretching vibrations [42,43,44] appeared in the FT-IR spectra of the CA-grafted Ln_2_O_3_ nanoparticles at 1556–1560 cm^−1^ and 1384–1389 cm^−1^, respectively, confirming successful grafting. Grafting resulted from hard acid‒hard base bonding between Ln^3+^ of Ln_2_O_3_ nanoparticles and COO^−^ groups of CA [45,46,47], while splitting arose from bridging COO^−^ groups bonded to Ln^3+^ on the nanoparticle surfaces [48]. In addition, the H‒C‒H antisymmetric (2966 cm^−1^) and symmetric (2922 cm^−1^) stretching vibrations of CA also appeared in the FT-IR absorption spectra of CA-grafted Ln_2_O_3_ nanoparticles, confirming successful CA grafting. The water H‒O‒H antisymmetric (3446 cm^−1^) and symmetric (~3257 cm^−1^) stretching vibrations were also observed in all samples as well as CA. The FT-IR absorption frequencies are summarized in Table 2.

Surface-grafting amounts (P) of CA, measured by TGA in wt.%, were 41.4, 43.3, and 43.8% for CA-grafted Ln_2_O_3_ nanoparticles (Ln = Eu, Gd, and Tb), respectively, after correcting for water and air desorption below ~105 °C (Figure 5b, Table 1). The residual TGA masses corresponded to the net Ln_2_O_3_ nanoparticle masses without CA. The grafting density (σ), defined as the average number of CA molecules per nanoparticle surface area [49], was 4.1, 3.9, and 4.9 for CA-grafted Ln_2_O_3_ nanoparticles (Ln = Eu, Gd, and Tb), respectively, using bulk densities of Eu_2_O_3_ (7.42 g/cm^3^), Gd_2_O_3_ (7.407 g/cm^3^), and Tb_2_O_3_ (7.91 g/cm^3^) [50], the d_avg_ from HRTEM, and TGA-derived *p* values. The average number (N_NP_) of CA molecules per nanoparticle was calculated by multiplying σ by the nanoparticle surface area (=πd^2^_avg_). High N_NP_ values indicate sufficient CA grafting for all nanoparticle samples, essential for colloidal stability and low toxicity, as confirmed in this study.

Based on FT-IR absorption spectra, Figure 5c illustrates the proposed CA surface grafting structure on nanoparticles using one representative CA molecule. TGA data shows that 40‒70 CA molecules were grafted per nanoparticle (Table 1).

### 2.4. In Vitro Cytotoxicity

**Figure 6:**
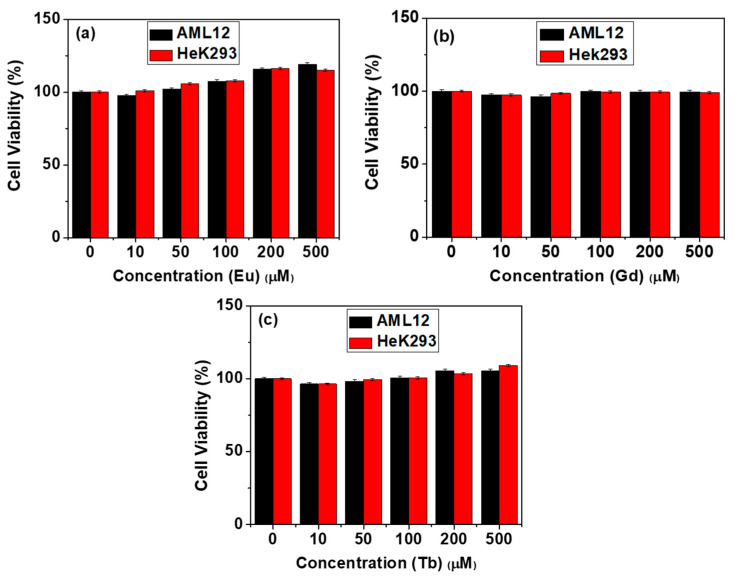
In vitro cell viability of AML12 and Hek293 cells after incubation with CA-grafted Ln_2_O_3_ nanoparticles: Ln = (**a**) Eu, (**b**) Gd, and (**c**) Tb.

The cellular cytotoxicity of CA-grafted Ln_2_O_3_ nanoparticles (Ln = Eu, Gd, and Tb) was assessed by measuring in vitro viability of human embryonic kidney 293 (Hek293) and normal mouse hepatocyte (AML12) cells after 48 h of incubation. All samples exhibited low cytotoxicity up to 500 μM [Ln] (Figure 6a–c).

### 2.5. Magnetic Resonance Imaging Properties

**Figure 7:**
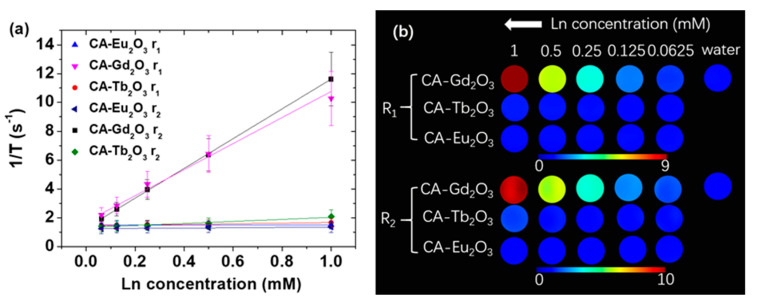
(**a**) Plots of 1/T_1_ and 1/T_2_ versus Ln concentration (Ln = Eu, Gd, and Tb) at H = 3.0 T. The slopes represent r_1_ and r_2_ values, respectively. (**b**) R_1_ and R_2_ map images as a function of Ln concentration.

The MRI contrast efficacy of the nanoparticles was assessed by measuring longitudinal (r_1_) and transverse (r_2_) water proton spin relaxivities, as well as longitudinal (R_1_) and transverse (R_2_) map images at H = 3.0 T. The r_1_ and r_2_ values were estimated from the plots of inverse longitudinal (1/T_1_) and transverse (1/T_2_) water proton relaxation times versus Ln concentration (Ln = Eu, Gd, and Tb) (Figure 7a, Table 3). CA-grafted Eu_2_O_3_ nanoparticles exhibited negligible r_1_ and r_2_ values owing to the low magnetic moment of Eu^3+^ (^7^F_0_) [18]. In contrast, CA-grafted Gd_2_O_3_ nanoparticles showed r_1_ and r_2_ values of 9.04 and 10.33 s^−1^mM^−1^, respectively, approximately twice higher than those of commercial Gd-chelates [51,52], indicating their potential as T_1_ MRI contrast agents given their r_2_/r_1_ ratio is close to one. This is attributed to the high density of Gd^3+^ (^8^S_7/2_) with a large 4f electron spin magnetic moment (s = 7/2) [18] per nanoparticle. For CA-grafted Tb_2_O_3_ nanoparticles, the r_1_ value was tiny because of 4f electron orbital motion contribution to the magnetic moment of Tb^3+^ (^7^F_6_) [52], while the small r_2_ value was because of the moderate magnetic moment of densely packed Tb^3+^ ions in nanoparticles. These properties suggest their potential as T_2_ MRI contrast agents at high MR fields (>3 T), where r_2_ is expected to increase [53]. By comparison, superparamagnetic iron oxide nanoparticles, such as Ferumoxytol (r_1_ = 13–25 s^−1^mM^−1^ and r_2_ = 150–350 s^−1^mM^−1^) [54], are well-known T_2_ MRI contrast agents owing to their high r_2_ values and large r_2_/r_1_ ratios. However, paramagnetic nanoparticles such as Ln_2_O_3_ (Ln = Dy, Tb, and Ho) nanoparticles are considered potential T_2_ MRI contrast agents at high MR fields owing to their r_2_ values which increase with MR field strength and tiny r_1_ values (<0.5 s^−1^mM^−1^) [53], enabling them to selectively enhance transverse water proton spin relaxations.

As provided in Table 3, CA-grafted Eu_2_O_3_ and Tb_2_O_3_ nanoparticles exhibited similarly low r_1_ values but lower r_2_ values than their D-glucuronic acid-grafted counterparts [22,53]. In contrast, CA-grafted Gd_2_O_3_ nanoparticles showed a higher r_1_ but a lower r_2_ value than those grafted with D-glucuronic acid [22]. These differences could be attributed to slight variations in particle size and ligands.

R_1_ and R_2_ map images help evaluate the potential of a material as an MRI contrast agent. CA-grafted Gd_2_O_3_ nanoparticles exhibited strong dose-dependent contrast enhancements in both map images, indicating their suitability to function as T_1_ or T_2_ MRI contrast agents, especially as T_1_ MRI contrast agents, owing to an r_2_/r_1_ ratio close to one (Figure 7b) [51]. In contrast, CA-grafted Eu_2_O_3_ nanoparticles displayed negligible dose-dependent contrast enhancements in the R_1_ and R_2_ map images, reflecting their minimal r_1_ and r_2_ values and unsuitability as contrast agents. CA-grafted Tb_2_O_3_ nanoparticles exhibited modest dose-dependent R_2_ contrast enhancements owing to a small r_2_ value, suggesting potential as T_2_ MRI contrast agents at high MR fields (>3 T) [53].

### 2.6. X-Ray Imaging Properties

**Figure 8:**
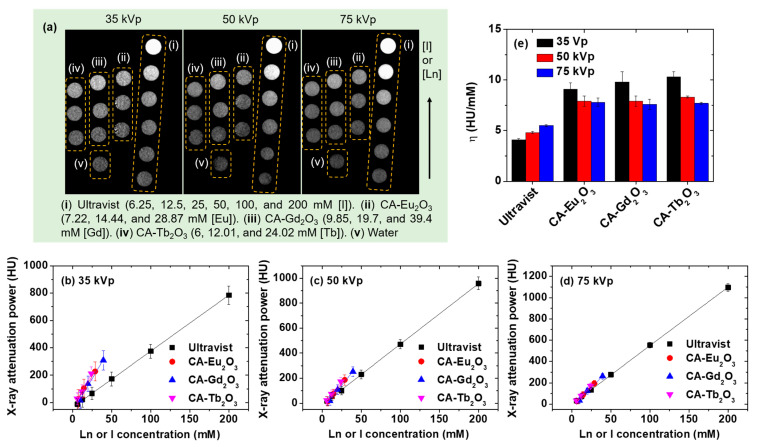
(**a**) Phantom images of CA-grafted Ln_2_O_3_ (Ln = Eu, Gd, and Tb) nanoparticles, Ultravist, and water at three X-ray voltages. Plots of X-ray attenuation versus atomic concentration [I] or [Ln] at (**b**) 35, (**c**) 50, and (**d**) 75 kVp. (**e**) Plots of X-ray attenuation efficiencies (η).

The X-ray phantom images were acquired at 35, 50, and 75 kVp to estimate X-ray attenuation of CA-grafted Ln_2_O_3_ (Ln = Eu, Gd, and Tb) nanoparticles dispersed in water (Figure 8a). Phantom images of water and Ultravist served as references. Plots of X-ray attenuation power in Hounsfield units (HU) extracted from the phantom images showed that all samples exhibited higher X-ray attenuation than Ultravist at equivalent atomic concentrations (Figure 8b‒d). This is because of the higher atomic numbers of Eu, Gd, and Tb (63, 64, and 65) compared to iodine (53) [56].

The X-ray attenuation efficiency (η) of the nanoparticles as well as Ultravist was estimated from the slopes (Figure 8b‒d) and listed in Table 3. At all X-ray voltages, the nanoparticles exhibited η values approximately two times higher than those of Ultravist (Table 3, Figure 8e), highlighting their potential as CT contrast agents. Additionally, compared to other studies [55], the η values of CA-grafted Ln_2_O_3_ nanoparticles were higher or similar compared to PAA-grafted Gd_2_O_3_ nanoparticles and PAA-grafted GdF_3_ nanoplates, confirming their potential as CT contrast agents.

## 3. Materials and Methods

### 3.1. Chemicals

GdCl_3_∙6H_2_O (99.9%), Tb(NO_3_)_3_∙5H_2_O (99.9%), Eu(NO_3_)_3_∙5H_2_O (99.9%), oleylamine (70%), sodium citrate tribasic dihydrate (CA) (99.0%), and dialysis tube [molecular weight cut-off (MWCO) = ~500 amu] were purchased from Sigma-Aldrich (St. Louis, MO, USA). Ethanol (>99%), hexane (>99%), and acetone (99%) were purchased from Duksan, Ansan, Republic of Korea. All chemicals were used as received. Triple-distilled water was used for washing the nanoparticles and preparing aqueous suspensions.

### 3.2. Synthesis of Ln_2_O_3_ (Ln = Eu, Gd, and Tb) Nanoparticles via Thermal Decomposition

**Figure 9:**
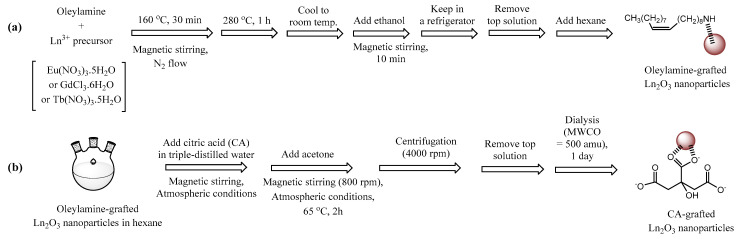
(**a**) Thermal decomposition synthesis of oleylamine-grafted Ln_2_O_3_ nanoparticles and (**b**) ligand exchange of oleylamine with CA to obtain CA-grafted Ln_2_O_3_ nanoparticles (Ln = Eu, Gd, and Tb).

Ln_2_O_3_ (Ln = Eu, Gd, and Tb) nanoparticles were synthesized by thermal decomposition (Figure 9a). About 2 mmol of Ln^3+^ precursors [Eu(NO_3_)_3_∙5H_2_O, GdCl_3_∙6H_2_O, or Tb(NO_3_)_3_∙5H_2_O] were added to 20 mL of oleylamine in a three-neck round bottom flask under N_2_ flow and heated to 160 °C for 30 min with magnetic stirring to remove water. Subsequently, the solution was heated to 280 °C at 5 °C/min and maintained for 1 h. After cooling to room temperature, the mixture was transferred to a 500 mL beaker, stirred with 400 mL of ethanol for 10 min, and preserved in a refrigerator until the oleylamine-grafted Ln_2_O_3_ nanoparticles settled. The supernatant was removed, and the nanoparticles were redispersed in 20 mL hexane.

### 3.3. Synthesis of CA-Grafted Ln_2_O_3_ Nanoparticles via Ligand Exchange

CA (5 mmol) was dissolved in 40 mL of triple-distilled water in a three-neck round bottom flask and mixed with 20 mL oleylamine-grafted Ln_2_O_3_ nanoparticles dispersed in hexane (Figure 9b). Thereafter, 15 mL of acetone was added and magnetically stirred at 800 rpm and 65 °C for 12 h under atmospheric conditions. After centrifugation, the aqueous phase (water and CA-grafted Ln_2_O_3_ nanoparticles) was separated by removing top organic phase (hexane and oleylamine). The product was dialyzed (MWCO = ~500 amu) against 1 L of triple-distilled water for 1 day to remove free CA, hexane, and acetone.

### 3.4. General Characterization

Nanoparticle diameter was measured by HRTEM (Titan G2 ChemiSTEM CS Probe; FEI, Hillsboro, OR, USA) at 200 kV after dispersing nanoparticles on a carbon film supported by a 200-mesh copper grid (Pelco No. 160, Ted Pella Inc., Redding, CA, USA). Elemental analysis was performed using an EDS instrument (Quantax Nano, Bruker, Berlin, Germany) inside the HRTEM.

Ln (Eu, Gd, or Tb) concentrations in aqueous nanoparticle suspensions were determined by inductively coupled plasma-atomic emission spectrometer (IRIS/AP, Thermo Jarrell Ash Co., Waltham, MA, USA).

Hydrodynamic diameter (a) and the zeta potential (ζ) of nanoparticle colloids (0.05 mM [Ln], Ln = Eu, Gd, and Tb) dispersed in aqueous media were measured using a DLS analyzer (Zetasizer Nano ZS, Malvern, UK).

The crystallinity of nanoparticle powder samples was characterized using a multipurpose XRD instrument (X’PERT PRO MRD, Philips, The Netherlands) with unfiltered CuKa (λ = 0.154184 nm) radiation, using a 0.033° scanning step over a 2θ scan range of 15°–100°.

CA attachment to Ln_2_O_3_ nanoparticles was analyzed by FT-IR absorption spectroscopy (Galaxy 7020A, Mattson Instrument Inc., Madison, WI, USA) on KBr pelletized powder samples over 400–4000 cm^−1^.

A TGA instrument (SDT-Q600, TA Instrument, New Castle, DE, USA) was used to measure CA grafting by heating powder samples from room temperature to 900 °C under air flow. After TGA, the remaining samples were subjected to XRD analysis for identification. The average CA content (wt.%) was estimated from mass loss after water and air desorption below ~105 °C, with the Ln_2_O_3_ amount estimated from the remaining mass.

### 3.5. MRI Imaging Parameters

T_1_ and T_2_ water proton spin relaxation times and R_1_ and R_2_ map images of CA-grafted Ln_2_O_3_ nanoparticles (Ln = Eu, Gd, and Tb) in aqueous media were measured using a 3.0 T MRI scanner (Magnetom Trio Tim, Siemens, Munich, Bayern, Germany). Aqueous solutions at 1, 0.5, 0.25, 0.125, and 0.0625 mM [Ln] were prepared by diluting the original concentrated samples with triple-distilled water. T_1_ relaxation was measured via inversion recovery, which varies inversion time (TI) over 35 TI values in the range of 50–1750 ms. T_1_ relaxation times were obtained by nonlinear least-square fitting of signal intensities at various TI values. T_2_ relaxation times were measured using the Carr–Purcell–Meiboom–Gill pulse sequence with 34 images acquired at 34 echo times (TE) ranging from 10 to 1900 ms and fitted similarly to mean pixel values. r_1_ and r_2_ values were then estimated from the slopes of inverse T_1_ and T_2_ versus Ln concentration plots, respectively.

### 3.6. X-Ray Imaging Parameters

X-ray phantom images of the CA-grafted Ln_2_O_3_ nanoparticles (Ln = Eu, Gd, and Tb) dispersed in aqueous media were acquired using a micro-CT scanner (Inveon, Simens Healthcare, Erlangen, Germany). Water and the commercial iodine CT contrast agent Ultravist were also imaged for comparison. X-ray attenuation power (HU) was estimated from these images. Measurement used X-ray source voltages/currents of 35 kVp/500 μA, 50 kVp/500 μA, and 75 kVp/500 μA, with a 170 ms imaging time per frame.

### 3.7. In Vitro Cellular Cytotoxicity Measurements

The in vitro cytotoxicity of the CA-grafted Ln_2_O_3_ nanoparticles (Ln = Eu, Gd, and Tb) was assessed using a CellTiter-Glo Luminescent Cell Viability Assay (Promega, Madison, WI, USA). Intracellular adenosine triphosphate was quantified using a Victor 3 luminometer (Perkin Elmer, Waltham, MA, USA). Hek293 and AML12 cells were seeded separately in 96-well plates and incubated for 24 h (1.5 × 10^4^ cells/well for AML12, 2 × 10^4^ cells/well for Hek293, 500 μL/well, 5% CO_2_, and 37 °C). Five test solutions (0.01, 0.05, 0.1, 0.2, 0.5 mM Ln, Ln = Eu, Gd, and Tb) were prepared by diluting the original concentrated nanoparticles dispersed in triple-distilled water with sterile phosphate-buffered saline (PBS) solution. Cells were treated with 2 μL aliquots and incubated for 48 h. Cell viability, measured in triplicate, was averaged and normalized to untreated control cells (PBS only, 0.0 mM Ln).

## 4. Conclusions

We successfully synthesized Ln_2_O_3_ nanoparticles (Ln = Eu, Gd, and Tb) via thermal decomposition and grafted them with hydrophilic and biocompatible CA through ligand exchange. The CA-grafted Ln_2_O_3_ nanoparticles were ultrasmall (2 nm), monodispersed, exhibited excellent colloidal stability in aqueous media, and had low in vitro cellular toxicity. Notably, the CA-grafted Gd_2_O_3_ nanoparticles had an r_1_ value approximately twice that of commercial Gd-chelates such as Gadovist and an r_2_/r_1_ ratio near one, indicating their potential as T_1_ MRI contrast agents. CA-grafted Tb_2_O_3_ nanoparticles exhibited low r_1_ and moderate r_2_ values, indicating their potential as T_2_ MRI contrast agents at high MR fields (>3 T), while CA-grafted Eu_2_O_3_ nanoparticles had negligible r_1_ and r_2_ values, making them unsuitable for MRI. In addition, all CA-grafted Ln_2_O_3_ nanoparticles exhibited η values approximately two times higher than that of the commercial iodine contrast agent Ultravist, indicating their potential as CT contrast agents.

## Figures and Tables

**Table 1:** Physicochemical properties of CA-grafted Ln_2_O_3_ nanoparticles (Ln = Eu, Gd, and Tb).

Nanoparticle	d_avg_ (nm)	a_avg_ (nm)	ζ (mV)	Surface-Grafting Amount		
				P ^a^ (wt%)	σ ^b^ (nm^−2^)	N_NP_^c^
Eu_2_O_3_	2.1 ± 0.1	26.4 ± 1.0	−16.7 ± 0.4	41.4	4.1	58
Gd_2_O_3_	1.9 ± 0.1	23.7 ± 1.0	−15.9 ± 0.2	43.3	3.9	44
Tb_2_O_3_	2.1 ± 0.1	27.7 ± 1.0	−11.0 ± 0.3	43.8	4.9	69

^a^ Average amount of CA molecules grafting a nanoparticle (in wt%). ^b^ Grafting density, i.e., average number of CA molecules grafting a nanoparticle unit surface area. ^c^ Average number of CA molecules grafting a nanoparticle.

**Table 2:** Summary of observed FT-IR absorption frequencies (cm^−1^).

Vibration ^a^	CA	CA-Eu_2_O_3_	CA-Gd_2_O_3_	CA-Tb_2_O_3_
(COO^−^) _AS_	1582	1556	1560	1556
(COO^−^) _SS_	1388	1384	1389	1384
(CH_2_) _AS_	2966	~2966	~2966	~2966
(CH_2_) _SS_	2922	2922	2922	2922
(H_2_O) _AS_	3446	~3318	~3318	~3318
(H_2_O) _SS_	~3257			

^a^ The subscripts, AS and SS indicate asymmetric stretching and symmetric stretching, respectively.

**Table 3:** r_1_, r_2_, and η values of CA-grafted Ln_2_O_3_ nanoparticles (Ln = Eu, Gd, and Tb) compared with those from other studies.

Material	d_avg_ (nm)	r_1_ (s^−1^mM^−1^)	r_2_ (s^−1^mM^−1^)	η (HU/mM)			Ref.
				35 kVp	50 kVp	75 kVp	
CA-Eu_2_O_3_	2.1	0.02 ± 0.01	0.11 ± 0.01	9.1 ± 0.6	7.9 ± 0.2	7.8 ± 0.4	This study
CA-Gd_2_O_3_	1.9	9.04 ± 0.53	10.33 ± 0.14	9.8 ± 1.0	7.9 ± 0.5	7.6 ± 0.5	This study
CA-Tb_2_O_3_	2.1	0.22 ± 0.03	0.71 ± 0.09	10.3 ± 0.5	8.3 ± 0.1	7.7 ± 0.1	This study
D-glucuronic acid-Eu_2_O_3_	2.0	0.006	3.82	-	-	-	[22]
D-glucuronic acid-Gd_2_O_3_	2.4	4.25	27.11	-	-	-	[22]
D-glucuronic acid-Tb_2_O_3_	2.0	<1.0	7.68	-	-	-	[53]
PAA ^1^-Gd_2_O_3_	1.9	-	-	5.9 (70 kVp)			[55]
PAA ^1^-GdF_3_ nanoplate	10.6 × 7.0 × 4.2 ^2^	-	-	~7.9 (60 kVp)			[55]
Ultravist	-	-	-	4.1 ± 0.1	4.8 ± 0.1	5.5 ± 0.1	This study
Gadovist	-	4.4 ± 0.1	4.7 ± 0.1	-	-	-	[51]

^1^ PAA = polyacrylic acid (Mw = ~1800 amu). ^2^ Nanoplate size = diameter × length × thickness.

## Data Availability

The original contributions presented in this study are included in the article.
